# Cancer stem cells and cisplatin-resistant cells isolated from non-small-lung cancer cell lines constitute related cell populations

**DOI:** 10.1002/cam4.291

**Published:** 2014-06-25

**Authors:** Blanca D Lopez-Ayllon, Veronica Moncho-Amor, Ander Abarrategi, Inmaculada Ibañez de Cáceres, Javier Castro-Carpeño, Cristobal Belda-Iniesta, Rosario Perona, Leandro Sastre

**Affiliations:** 1 Instituto de Investigaciones Biomédicas CSIC/UAM, Biomarkers and Experimental Therapeutics in Cancer, IdiPAZSpain; 2 Instituto de Salud Carlos III, Unidad de Biotecnología CelularSpain; 3 Cancer Epigenetics Laboratory, INGEMM, University Hospital La Paz, Biomarkers and Experimental Therapeutics in Cancer, IdiPAZSpain; 4 Department of Medical Oncology, University Hospital La PazSpain; 5 Department of Medical Oncology, University Hospital Madrid Norte-SanchinarroSpain; 6 CIBER de Enfermedades RarasValencia, Spain

**Keywords:** Cancer stem cells, cancer-initiating cells, cisplatin resistance, lung cancer, NSCLC

## Abstract

Lung cancer is the top cause of cancer-related deceases. One of the reasons is the development of resistance to the chemotherapy treatment. In particular, cancer stem cells (CSCs), can escape treatment and regenerate the bulk of the tumor. In this article, we describe a comparison between cancer cells resistant to cisplatin and CSCs, both derived from the non-small-cell lung cancer cell lines H460 and A549. Cisplatin-resistant cells were obtained after a single treatment with the drug. CSCs were isolated by culture in defined media, under nonadherent conditions. The isolated CSCs were clonogenic, could be differentiated into adherent cells and were less sensitive to cisplatin than the original cells. Cisplatin resistant and CSCs were able to generate primary tumors and to metastasize when injected into immunodeficient Nu/Nu mice, although they formed smaller tumors with a larger latency than untreated cells. Notably, under appropriated proportions, CSCs synergized with differentiated cells to form larger tumors. CSCs also showed increased capacity to induce angiogenesis in Nu/Nu mice. Conversely, H460 cisplatin-resistant cells showed increased tendency to develop bone metastasis. Gene expression analysis showed that several genes involved in tumor development and metastasis (EGR1, COX2, MALAT1, AKAP12, ADM) were similarly induced in CSC and cisplatin-resistant H460 cells, in agreement with a close similarity between these two cell populations. Cells with the characteristic growth properties of CSCs were also isolated from surgical samples of 18 out of 44 lung cancer patients. A significant correlation (*P* = 0.028) was found between the absence of CSCs and cisplatin sensitivity.

## Introduction

Lung cancer represents a life-threatening disease that produced 22.3% of the cancer-related deceases in men and 11.3% in women worldwide in 2008 1. Besides its high incidence, mortality is increased by the frequent development of resistance to the drugs used in the treatment. Relapses often appear after surgical or pharmacological treatment that are more resistant to chemotherapy than the original tumor 2. Therefore, a large effort is being devoted to the study of lung cancer, its development and the acquisition of drug resistance.

One model that has received considerable attention in last years explains the existence of relapses and the acquisition of resistance to chemotherapy by the existence of Cancer Initiating Cells, also designed as cancer stem cells (CSCs) 3. According to this model, a proportion of the tumor cells are initiating cells that divide asymmetrically to generate a new stem cell and a daughter cell that differentiates and also continues proliferating to contribute to the main mass of the tumor. Stem cells would be more resistant to chemotherapy and would more probably overcome tumor treatment. Therefore, remaining stem cells would be able to regenerate the tumor, producing relapses that could acquire increased resistance to the drug used for primary tumor treatment.

Cancer stem cells have been identified in different tumors. Their identification can be based on the expression of specific markers, on the capacity of these cells to secrete fluorescent molecules or on their capacity to grow in defined media under nonadherent conditions. The more frequent type of lung cancer (85% of the cases) is non-small-cell lung cancer (NSCLC). Several articles have reported the isolation of CSC from NSCLC cell lines (reviewed in [4, 5]). In most of these studies the expression of different stem cells markers or fluorescent dyes exclusion criteria were used for isolation of the stem cells.

Drug resistance has also been studied in NSCLC cells. One of the chemotherapeutic drugs more extensively used for NSCLC treatment is cisplatin and several authors, including ourselves 6, have studied cisplatin resistance in lung cancer. Among the mechanisms described are alterations of intracellular signaling pathways, changes in RNA expression or epigenetic mechanisms (for recent reviews 7,8).

In this article, we have approached the possible relationship between induced cisplatin resistance and the stem properties of NSCLC cells. Cancer stem cells were isolated because of their capacity to grow in a defined culture media, under nonadherent conditions. On the other hand, cisplatin-resistant cells were obtained after a single treatment with cisplatin, in an attempt to reproduce the protocols used for patient chemotherapy. Both cell populations have been compared in terms of cisplatin sensitivity, tumorigenicity, metastatic capacity, and gene expression profiles and several similarities have come out, in agreement with the CSC model.

## Materials and Methods

### Cell culture, transfection, and selection of cisplatin resistant and CSC populations

Human NSCLC H460 and A549 cells, purchased from the American Type Culture Collection were cultured in RPMI (Roswell Park Memorial Institute, Gibco, Carlsbad, CA) media supplemented with 10% foetal bovine serum (FBS). Cisplatin-resistant cells were obtained after treatment of H460 or A549 cells with 0.5 *μ*g/mL (1.7 *μ*mol/L) or 2.5 *μ*g/mL (8.3 *μ*mol/L) of cisplatin, respectively, for 72 h. After this treatment, cells were cultured in the absence of the drug. Cancer stem cell populations were isolated by culture in DMEM (Dulbeco modified eagle medium)/F12 (Gibco, Carlsbad, CA) (1:1) media supplemented with 2 mmol/l-Glutamine, 5 mmol/L Hepes, 0.4% BSA (bovine serum albumin), N2 supplement (Gibco, Carlsbad, CA) and 20 ng/mL of epidermal growth factor and bFGF (basic fibroblast growth factor) (PeproTech, Rocky Hill, NJ). Cells were transfected with the plasmid pmCherry-N1 using Lipofectamine 2000 transfection reagent (Invitrogen, Carlsbad, CA) and selected by their resistance to neomycin (G418). Green-Fluorescent protein (GFP)-expressing cells were obtained by infection with the pGIPZ-shRNA^mir^-NS lentiviral vector (Open Biosystems, Thermo Scientific, Madrid, Spain).

### Cell-viability assays

Cells were cultured in 96-well plates at a density of 50 cells/well for 24 h, cisplatin was added and the culture continued for 72 additional hours. The number of viable cells was estimated using the MTS ([3-(4,5-dimethylthiazol-2-yl)-5-(3-carboxymethoxyphenyl)-2-(4-sulfophenyl)-2H-tetrazolium) hydrolysis method (Promega Corporation, Madison, WI).

### Clonogenicity assays

The capacity of the cells to grow as clones derived from single cells was assayed by soft-agar culture, as described 9. Clonogenicity was also tested in liquid culture by seeding individual cells in 96-wells plates under adherent and nonadherent culture conditions.

### In vitro cell invasion assays

10^4^ cells were seeded in the upper part of BD BioCoat™ Matrigel™ invasion chambers (BD Biosciences, San Jose, CA) in medium containing 0.5% FBS, 0.1% BSA. In the case of CSCs, defined media containing 0.1% BSA and without growth factors was used. Culture medium containing 10% FBS was added to the lower part of the chambers. Cells that had invaded the matrigel layer after 24 h of culture were stained using the Diff Quick method (Medion Diagnostics, Duedingen, Switzerland) and quantified using the analySIS program (Soft Imaging System Olympus, Tokyo, Japan).

### Tumorigenicity in xenograft mouse models

Matrigel (BD Biosciences)-impregnated cells were injected in both flanks of athymic Foxn1^nu^ female mice. Tumor volume was calculated as l × w^2^ × 0.52, being l the length and w the width of the tumors. In cell-mixture experiments, cells expressing the GFP or the Cherry proteins were mixed before injection into mice. Mice were sacrificed 30 days later tumors were removed, fixed in formaldehyde, included in OCT (Optimal Cutting temperature), and cut in 10 *μ*m sections. The percentage of GFP and Cherry-expressing cells was determined using the ImageJ program (National Institutes of Health, Bethesda, MD).

### In vivo angiogenesis assays

Athymic female mice were subcutaneously injected with 300 *μ*L of Matrigel containing 30 *μ*g of conditioned media. bFGF and PBS (phosphate buffered saline) were used as positive and negative controls, respectively. Mice were sacrificed 10 days after injection, plugs were removed and the presence of microvessels assayed by CD31 expression as previously described 10.

### Metastasis assays

Nude mice of 6 weeks were injected in the tail with 10^6^ cells suspended in 100 *μ*L of physiologic serum. After animal's death, a complete necropsy examination was performed 11. The brain, salivary glands, and visceral organs were fixed in 4% formaldehyde, embedded in paraffin and cut in 5 *μ*m sections that were stained with Hematoxylin and Eosin. To study bone metastasis, femurs were fixed with 4% formaldehyde for 24 h and incubated in 70% ethanol at 4°C before decalcification in 4% HCl, 4% formic acid for 5 days. Bones were then incubated in 70% ethanol and embedded in paraffin. Five *μ*m sections were obtained and stained with Hematoxylin and Eosin.

### Gene expression analyses by microarray hybridization

Total cellular RNA was isolated using the Trizol reagent (Invitrogen, Carlsbad, CA) and purified with the RNeasy Mini kit (Qiagen, Valencia, CA). Duplicate RNA samples were converted to labeled cDNA using the Two-Color Microarray-Based Gene Expression Analysis kit (Quick Amp Labelling; Agilent Technologies, Santa Clara, CA). Dye-swap labeled cDNAs were hybridized to Whole Human Genome Microarrays 4x44K G4112F (Agilent) in technical duplicates. Hybridized Microarrays were scanned and the data extracted using the Feature Extraction Software (Agilent). Analysis of the data was performed at the National Biotechnology Center (CNB, Madrid, Spain). Differential expression comparisons were made using the Rank Products method 12. Data were filtered and visualized using the FIESTA program 13. Genes common in different comparisons were identified and represented in a Venn diagram using the VENNY program 14. Functional enrichment of differentially expressed genes was analyzed using the GOTree Machine (GOTM) 15.

### Quantitative RT-PCR analysis of gene expression

cDNA was obtained from 1 *μ*g of each RNA using the High-Capacity cDNA Archive Kit (Applied Biosystems, Madrid, Spain). Quantitative PCR analyses was carried out in triplicate samples using TaqMan® probes and the Taqman Universal PCR Master Mix (Applied Biosystems) using a Step One Plus Real-Time PCR System (Applied Biosystems). The following probes were used in these studies: Hs03929097_g1 (GAPDH), Hs99999903_m1 (*β*-actin), Hs01555410_m1 (IL1B), Hs00158757_m1 (LOXL2), Hs02562698_s1 (ADM), Hs00152928_m1 (EGR1), Hs01910177_s1 (MALAT1), Hs00153133_m1 (COX2), Hs1103582_s1 (JUN), Hs011112126_m1 (AKAP12), Hs02330069_s1 (CXCR4), Hs00610256_g1 (DUSP1), Hs00737962_m1 (DUSP6), Hs00153458_m1 (VEGFC), Hs03044178_g1 (CD24), Hs01053790_m1 (ABCG2). Relative gene expression quantification was calculated according to the comparative threshold cycle method 16 using *β*-actin or GAPDH as endogenous controls.

### Protein expression analysis

Cells extracts were obtained and analyzed as described 17. Primary antibodies were obtained from Santa Cruz Biotechnology (Santa Cruz Biotech, Dallas, TX) (Fibronectin), Cell Signaling (Cell Signalling Technology, Danvers, MA) (E-Cadherin, Vimentin, ZO-1) or Sigma (St. Louis, MO) (*α*-tubulin). Secondary antibodies were obtained from BioRad (Berkeley, CA) and Cell Signaling.

### Processing of surgical samples

Surgical samples of lung cancer were collected at the Hospital Universitario La Paz, Madrid, Spain. Samples were collected in DMEM/MixF12Ham (Sigma), digested with Collagenase (0.3 mg/mL), and Hyaluronidase (125 U/mL) (both from Sigma) for 20 min at 37°C, mechanically disaggregated and filtered.

## Results

### Isolation of cisplatin-resistant NSCLC cells

**Figure 1:**
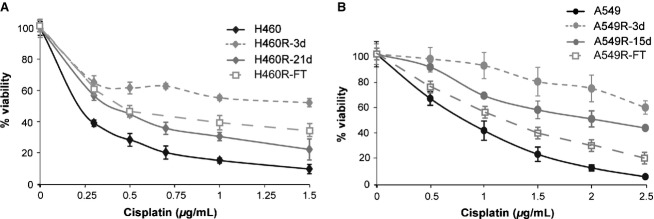
Cisplatin sensitivity of the NSCLC H460 and A549 cells after a single treatment with the drug. H460 and A549 cells were incubated with 0.5 *μ*g/mL (1.7 *μ*mol/L) or 2.5 *μ*g/mL (8.3 *μ*mol/L) of cisplatin, respectively, for 3 days. The cells that survived the treatment, H460R (A) and A549R (B), were assayed for cisplatin sensitivity either at the end of cisplatin treatment (H460R-3d, A549R-3d), at the time when treated cells reached confluence (H460-21d, A549R-15d) or after these cells were frozen and thaw out back into culture (H460R-FT, A549R-FT). Cell viability was determined using the MTS reagent. Average and Standard deviations of three independent experiments are shown.

H460 and A549 NSCLC cell lines were incubated with a single dose of cisplatin, 0.5 *μ*g/mL (1.7 *μ*mol/L) for H460 (determined IC50 = 0.3 *μ*g/mL) and 2.5 *μ*g/mL (8.3 *μ*mol/L) for A549 cells (determined IC50 = 0.75 *μ*g/mL). The possible induction of drug resistance was analyzed after 3 days in drug-free culture (R-3d), when treated cells reached confluence (21 days for H460 R-21d, 15 days for A549 R-15d) and after these cells were frozen and thaw out again into culture (H460R-FT, A549R-FT). Cells that survived to a single dose of cisplatin were less sensitive to this drug than the original populations (Fig.1). Sensitivity increased from 3 to 15 or 21 days in culture in the absence of drug and got stabilized for prolonged periods of culture thereafter.

### Isolation and characterization of CSCs from NSCLC cell lines

**Figure 2:**
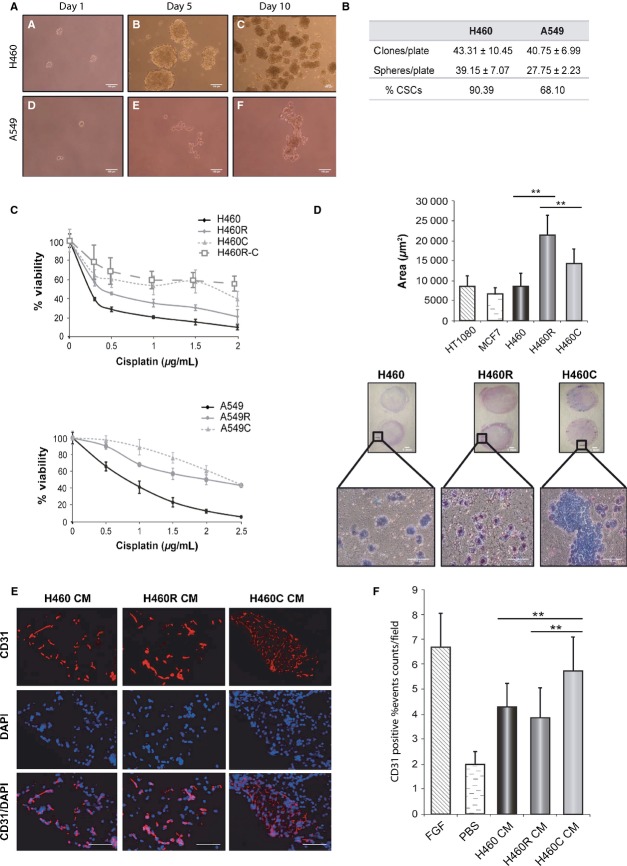
Isolation and characterization of cells with Cancer Stem Cells (CSC) characteristics from H460 and A549 cells. (A) H460 (A–C) and A549 (D–F) cells were cultured in defined media, under nonadhesive conditions for 1–10 days. Pictures were taken using a Nikon TS100 microscope. The bar represents a distance of 100 *μ*m. (B) H460 and A549 cells were cultured in 96 well plates at an average density of 48 cell/plate and the number of clones or spheres present after 10 days of culture was counted. The upper row represents the number of clones obtained culturing the cells in standard serum-containing media under adherent conditions. The middle row represents the total number of spheres containing more than four cells obtained culturing the cells in defined media and under nonadherent conditions. The percentage of spheres (%CSC), as compared to the number of clones, is represented in the lower row of the table. (C) Cisplatin sensitivity. H460 and A549 CSC (H460C, A549C), cisplatin resistant (H460R, A549R), CSCs isolated from H460-resistant cells (H460R-C) or untreated cells (H460, A549) were incubated with 0–2.5 *μ*g/mL of cisplatin for three days. The number of viable cells was estimated using the MTS reagent at the end of the treatment. Average values and standard deviations of three independent experiments are represented. (D) Migration of H460 untreated (H460), cisplatin-resistant (H460R) and CSC (H460C) cells through a matrigel cushion. Fetal Calf Serum was used as chemoatractant. HT1080 and MCF7 cells were used as controls. The lower panel shows microscopic images of the cells that migrated through the matrigel cushion. The quantification of the number of cells that migrated (ten fields for each cell population) is indicated on the upper panel. Significant differences between H460/H460R and H460R/H460C cells are indicated by asterisks (*P < 0.05, **P > 0.01). (E and F) Angiogenic capacity of H460 cisplatin-resistant and CSC cells. Conditioned media obtained for untreated (H460), cisplatin-resistant (H460R) of CSC (H460C) cell lines was embedded in matrigel and subcutaneously implanted in Nu/Nu mice. Matrigel plugs were extracted 10 days after implantation and the presence of endothelial cells analyzed by immunohistochemistry using anti-CD31 antibodies. Panel E shows microscopic pictures of the sections where CD31 expression is indicated in red and DAPI nuclear staining in blue. The lower pictures show the superposition of DAPI staining and CD31 expression. Panel F shows the quantification of CD31 staining. FGF was used as a positive control and the buffer PBS as a negative control. Significant differences between H460/H460C and H460R/H460C cells are indicated by asterisks (**P > 0.01).

H460 and A549 cells were cultured in defined, serum-free, media under nonadherent conditions to detect the possible presence of cells with tumor initiating characteristics. Cells from both cell lines were able to grow forming spheroid aggregates (Fig.2A). The clonogenic capacity was analyzed by plating them in 96-Well plates. An average number of 48 cells were seeded on each plate under adherent or nonadherent conditions. Figure2B indicates the total number of clones or spheres containing more than four cells obtained. Spheres were originated from individual cells that were considered CSCs.

The dependence of these cells on growth factors was determined. The results obtained (Fig. S1A) indicate that while A549 cells were dependent on the growth factors added to the media, H460 cells grew in their absence. Actually, conditioned media obtained from H460 cells supported A549 spheres growth without additional growth factors (Fig. S1A).

The expression of CSC markers was analyzed by quantitative reverse transcription-polymerase chain reaction (RT-PCR, Fig. S1B). H460 CSCs showed increased expression of CD133 and decreased levels of CD44 and CD166. In contrast, A549 CSCs showed increased levels of CD44 and decreased CD133 expression. H460 cisplatin-resistant cells showed increased CD133 expression, as H460C cells. A549 cisplatin-resistant cells showed increased CD44 expression, as A549C cells, but also increased CD133 expression (Fig. S1B). Both H460C and A549C cells expressed lower levels of the CD24 and ABCG2 CSC marker genes than untreated cells. H460R cells also expressed lower levels of both genes while A549R cells showed decreased CD24 and increased ABCG2 expression (data not shown).

CSCs are supposedly more resistant to anticancer drugs than the bulk of cells from the same tumor. The sensitivity to cisplatin of H460 and A549 CSCs was analyzed and both CSCs were less sensitive to the drug than untreated cells (Fig.2C). Actually, previously isolated resistant cells showed an intermediate behavior between CSCs and untreated cells (Fig.2C). CSCs isolated from H460-resistant cells (H460R-C) showed a cisplatin sensitivity similar to that of H460C cells (Fig.2C).

CSCs might also have increased invasive capacity and undergo Epithelial/Mesenchymal Transition (EMT). Both characteristics were analyzed for H460 CSCs and cisplatin-resistant cells. A significant increase in cell migration was observed for H460 cisplatin-resistant cells (H460R) using an in vitro Matrigel invasion assay (Fig.2D). H460 CSCs also showed increased migration although the difference with untreated H460 cells was not statistically significant. However, CSCs migrated as cell aggregates (Fig.2D) which might result in an underestimation of their migration capacity, determined as the surface of the filter covered by migrating cells.

Epithelial/Mesenchymal Transition was determined by studying the expression of epithelial and mesenchymal markers. CSCs, cisplatin-resistant cells and CSCs that were allowed to redifferentiate by culture in adherent plates with standard culture media (dif CSC) were analyzed. H460 and A549 cells differed markedly in the expression of epithelial and mesenchymal markers such as E-Cadherin, Fibronectin, ZO-1 or Vimentin (Fig. S1C, see expression ratios). However, both derived CSCs showed similar response, the expression of epithelial markers (E-cadherin, ZO-1) and one of the mesenchymal markers (Fibronectin) increased while the other mesenchymal marker (Vimentin) showed decreased expression. Differentiation of H460 CSCs reverted these changes (Fig. S1C). These results indicated a mixed epithelial/mesenchymal phenotype in the CSCs.

### Tumorigenic capacity of the H460 CSCs and cisplatin-resistant cells

Different numbers of untreated, H460 resistant or H460 CSCs were injected subcutaneously in immunodeficient mice. Latency and tumor size 30 days after cell injection was determined (Table S1). CSCs and resistant cells induced smaller tumors with a longer latency period, indicating that they grew more slowly than the untreated H460 cells in the mouse xenografts. Since tumors are considered a mixture of CSCs and differentiated cells, we analyzed the behavior of mixed populations of both cell types. H460 cells expressing the Cherry-fluorescent protein were mixed with CSCs expressing GFP to distinguish the cells of the tumor coming from each population. Labeled H460 and CSCs were mixed in different proportions before inoculation. The latency period and tumor size at the end of the experiment were determined (Table 1). Tumors were resected and the proportion of red-H460 and green-CSCs estimated. In three of the four cell mixtures tested, the proportion of CSCs at the end of the experiment was smaller than at the beginning, indicating slower growth, in agreement with the results obtained injecting CSCs alone. However, when 25% of CSCs were present, their percentage increased to 34.58% in the tumor. In addition, the tumors obtained showed shorter latency and larger volume than those obtained with other cell proportions. These data indicate that some proportions of CSCs and differentiated cells can potentiate the growth of both cell types, resulting in more aggressive tumors.

**Table 1:** Characteristics of the tumors formed after injection in Nu/Nu mice of mixtures, in different proportions, of H460 and H460 CSC (H460C) cells

Inoculated cells		Tumor distribution			
			
% H460	% H460C	% H460	% H460C	Latency (days)	Tumor volume (cm^3^)
95	5	∼99 ± 0.094	<1 ± 0.094	20 ± 2.18	0.868 ± 0.42
85	15	90.13 ± 2.42	9.87 ± 2.42	20 ± 4.3	0.748 ± 0.39
75	25	65.42 ± 3.44	34.58 ± 3.44	15 ± 3.42	2.5 ± 0.53
50	50	54.56 ± 10.61	45.44 ± 10.61	23 ± 3.1	0.943 ± 0.43

The proportion of each cell population in the generated tumors, their size and the latency period are indicated.

The angiogenic potential of H460, CSCs, and cisplatin-resistant cells was evaluated in vivo. Conditioned media prepared from these cell lines was included in matrigel plugs and implanted subcutaneously into flanks of nude mice. Plugs were extracted 10 days later and the presence of vascular endothelial cells determined by immunohistochemistry using anti-CD31 antibodies. Conditioned media from CSCs attracted significantly more endothelial cells than the one from H460 or cisplatin-resistant cells (Fig.2E and F). These results demonstrated that H460 CSCs promote new vessel formation more efficiently than the other cell lines.

Metastatic capacity was determined injecting the cells in the tail vein of immunodeficient mice. Mice were killed when they showed decreased motility and loss of weight and the formation of tumors was determined by anatomic examination of the organs and posterior histological analyses. H460, CSCs, cisplatin-resistant cells, and a combination of H460 and CSCs in equal proportions were studied. Most of the inoculated mice developed lung tumors, as expected from the inoculation protocol used (Table 2), but also developed metastatic tumors in other organs, as described for H460 cells 10. CSCs produced less tumors that H460 cells (Table 2; 1.4 vs. 1.9), but the combination of CSCs and H460 cells produced more metastasis than any of the two cell lines (2.25 tumor/mouse), indicating that they potentiate each other, in agreement with the tumorigenic studies.

**Table 2:** Metastatic tumors formed after intravenous injection of H460 cells, H460 CSC (H460C), H460 cisplatin-resistant cells (H460R) or a combination of H460 and H460 CSC (H460 + H460C) cells

Cell Line →	H460	H460C	H460 + H460C	H460R
Lung	8	12	4	11
Other organs				
Brain	–	–	–	–
Salivary gland	1	–	–	2
Trachea	–	2	1	–
Heart	–	1	1	–
Liver	1	–	–	–
Spleen	–	–	-	–
Pancreas	1	1	–	–
Intestine	–	1	–	–
Kidney	3	1	1	1
Genitals	2	–	–	1
Bone	1	–	1	5
Other tumors	4	3	1	6
Total number of mice	11	15	4	14
Metastasis/mouse	1.90	1.4	2.25	1.86

**Figure 3:**
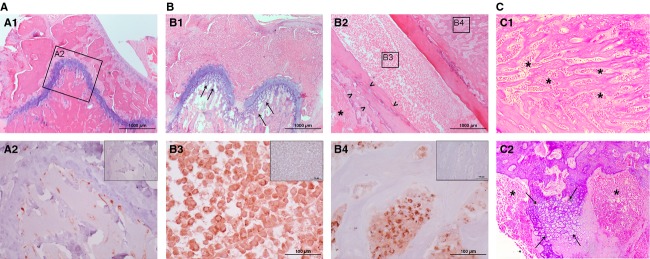
Bone metastasis to femur of, vein tail implanted, cisplatin-resistant H460 cells. (A) Femur sample in initial phase of bone metastasis. (A1) Hematoxylin and Eosin (H&E) staining shows healthy appearance. Immunohistochemistry (IHC) in square-detained area (A2) shows metastatic cells in metaphysis area and intimately related to blood vessels. (B) Femur sample in advanced metastasis phase. H&E Staining shows (B1) Metaphysis area with morphological changes in cartilage of growth plate (Arrows), (B2) Hepiphysis area with bone lysis (Arrow heads) in cortical bone and extramedular tumor mass (Star). IHC in square-detained areas show (B3) Bone marrow fully infiltrated with tumoral cells and (B4) Human cell-infiltrated reactive bone area. (C) Details of native tissue reaction to advanced metastasis. (C1) Reactive bone formation in epiphysis. (C2) Hypertrophic cartilage (Arrows) in metaphysis. Both areas are fully infiltrated with tumoral cells (asterisks). Inserted figures in A2, B3, and B4 correspond to negative controls.

Cisplatin-resistant cells produced a similar number of tumors than H460 cells (1.86 tumors/mouse) but a larger number of bone metastasis (5/14 vs. 1/11). Bilateral bone metastases were observed in most positive mice. Histological analysis of femurs indicated that metastatic precursors appeared preferably in metaphysis (Fig.3A). Figure 3A2 shows some metastatic cells in the bone marrow and next to growth plate, intimately related to blood vessels. Samples with more evident traces of metastasis (Fig.3B–C) showed complete colonization of the bone marrow and hypertrophic cartilage tissue in growth plate area. Also bone tissue integrity was compromised, with destruction of cortical bone and reactive trabecular bone formation. Reactive new bone was formed by native bone cells and it appeared fully infiltrated by tumoral cells (Fig.3B4). Large bone lytic areas and extraosseous tumor-mass formation were observed in the more metastasis-advanced samples. These data indicate that metastasis mainly starts in bone marrow of metaphysis area and later affect whole bone structure (Fig. 3).

### Gene expression profiles

The experiments shown above indicated some similarities between H460 CSCs and cisplatin-resistant cells. To further characterize these cell populations, their gene expression profile was analyzed by DNA microarray hybridization. Agilent human DNA microarrays were hybridized to cDNAs derived from H460, CSCs or cisplatin-resistant cells (GEO code GSE54981). Differences in gene expression, in comparison to H460 cells, were identified using a False Discovery Rate (FDR) smaller than 0.05. A summary of the more relevant genes detected is shown in Table S2. Genes differentially regulated in CSCs and cisplatin-resistant cells were compared. A total of 13 genes were commonly upregulated and 13 downregulated in both cells, in comparison to H460 cells (Fig. S2). Commonly upregulated genes whose function can be more relevant in cancer-cell biology are shown in Table 3.

**Table 3:** Summary of the expression levels determined by DNA microarray analyses comparing H460 cells to cisplatin-resistant H460 cells (H460R), CSC H460 cells (H460C)

Gene	Accession number	Description	Fold change (H460R)	Fold change (H460C)	Fold change (H460CDif)
EGR1	NM_001964	*Early growth response 1*	38.08	12.78	–
JUN	NM_002228	*Proto-oncogene*	13.39	13.27	–
PTGS2/COX2	NM_000963	*Prostaglandin-endoperoxide synthase 2/Cyclooxygenase 2*	4.62	17.84	–
MALAT1	NR_002819	*Metastasis-associated lung adenocarcinoma transcript 1*	8.59	5.15	–
AKAP12	NM_144497	*Kinase (PRKA) anchor protein 12*	3.51	7.16	–
ADM	NM_001124	*Adrenomodullin*	5.13	5.6	−4.8
CXCR4	NM_001008540	*Chemokine (C-X-C motif) receptor 4*	–	8.02	−7.5
IL1B	NM_000576	*Interleukin 1, beta*	–	8.73	−5.1
LOXL2	NM_002318	*Lysyl oxidase-like 2*	–	6.72	−8.2

The right column compares CSC cells before and after culture under adherent conditions for 24 h (H460CDif).

The identification of genes differentially regulated in CSCs was also approached in a redifferentiation study where CSCs were cultured under nonadherent conditions for 10 days and then changed to adherent culture conditions in serum-containing media for 3, 9 or 24 h. Adherent cells appeared under these culture conditions. Gene expression was compared by DNA microarray hybridization (GEO code GSE54712). Differentially-regulated genes were selected using a FDR < 0.05. The results obtained are summarized in Table 3 and Table S3.

The differential expression of some of these genes was validated by quantitative RT-PCR (Fig. S3A). All the genes analyzed showed a significant induction in CSCs, as compared to H460 cells, and also a decrease in expression in differentiated CSCs. Four of the nine genes analyzed also showed increased expression in cisplatin-resistant H460 cells. Our group had previously described that DUSP1 controlled the expression of DUSP6 and proangiogenic genes such as VEGFC 10. Inhibition of DUSP6 and VEGFC expression correlated with decreased metastasis and angiogenic potential. Furthermore, DUSP1-depleted H460 cells showed increased sensitive to cisplatin 6. Therefore, we have determined the expression of these genes in the cells obtained in this study. The three genes were upregulated in H460 cisplatin- resistant and CSCs (Fig. S3B). In addition, the three genes were also upregulated in A549 cisplatin-resistant cells and two of them (DUSP6, VEGFC) in A549 CSCs, further enforcing the relation between the cisplatin resistance and CSC potential in NSCLC cell lines.

### Isolation of CSC cells from clinical samples

**Figure 4:**
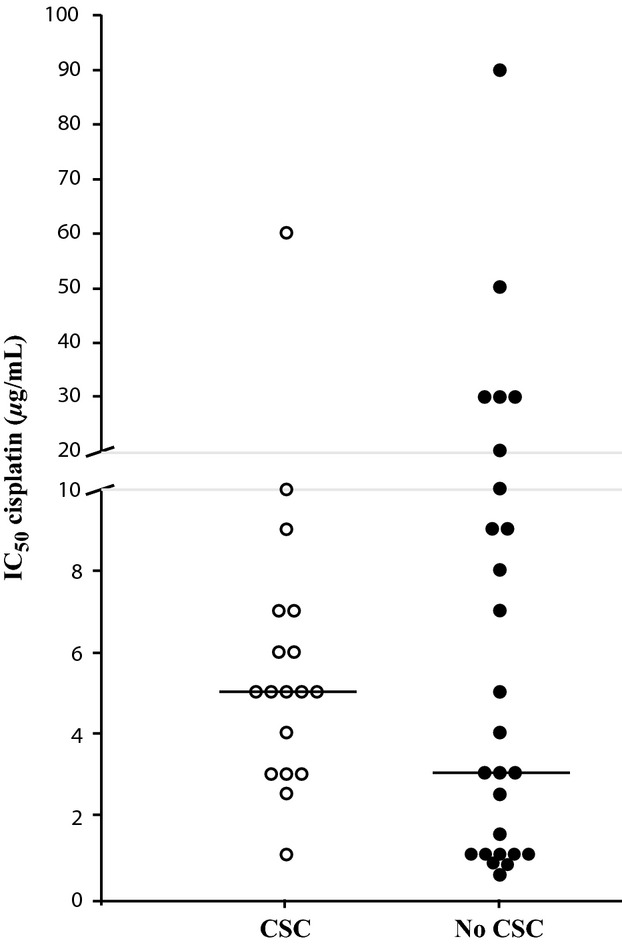
Comparison of cisplatin sensitivity and the presence of sphere-forming cells in surgical samples of NSCLC patients. Surgical samples of NSCLC patients that had not received any pharmacological treatment were disaggregated. Half of the cells obtained were used to analyze cisplatin sensitivity and their IC50 determined. The other half was cultured in defined media under nonadherent conditions and the presence of cell spheres determined after 30 days in culture. The IC50 values obtained are represented for each patient. Patient samples that contained sphere-forming cells are indicated with open circles (CSC) and those that did not with close circles (NO CSC). A IC50 value of 2 *μ*g/mL was considered the limit between cisplatin sensitive and resistant cells. Bar, median value of each group.

The presence of CSCs in clinical isolates and the possible correlation to cisplatin resistance was analyzed. Surgical samples were obtained from 44 patients diagnosed from NSCLC that had not received chemotherapy (patients' characteristics are described in Table S4). Samples were processed to isolate dispersed cells. One part of the cells was used to determine cisplatin sensitivity and the other part cultured under nonadherent conditions in defined media to observe the possible presence of CSCs. Figure4 shows the results obtained in the double assay. Samples able to form spheres are indicated with open circles and those were no spheres were observed after 30 days in culture with filled circles. The IC50 for cisplatin obtained from each sample is represented in the *Y*-axis.

Previous studies of multiple cell lines in our group indicated that the average IC50 for cisplatin is 2 *μ*g/mL. Taking this value as a reference, nine of the patients' samples that were sensitive to cisplatin were not able to form CSC spheres and only one sensitive sample formed them. These results would indicate that patients with a low capacity to form CSC spheres are more alike to be sensitive to cisplatin. The statistical test of this hypothesis using the chi square method gave a significant *P*-value of 0.028.

## Discussion

The biological characteristics and gene expression profiles of cisplatin resistant and CSCs isolated from two NSCLC cell lines have been compared. Cisplatin-resistant cells, isolated after a single treatment with the drug, showed reduced sensitivity to cisplatin even after prolonged periods of culture in the absence of the drug. These results are in agreement with previous studies 18 and indicate that cisplatin resistance can be induced in patients from the first treatment and can increase in successive rounds of treatment.

CSCs were isolated by culture in serum-free media under nonadherent conditions. This strategy has been previously used for isolation of CSCs from NSCLC established 19 and primary cell lines 20,21. The isolated populations present CSCs characteristics: self-renewal, differentiation capacity in the presence of serum, tumorigenicity, and increased drug resistance. Putative CSCs were also isolated from 40% of 44 patient samples analyzed using the same culture conditions. Several authors have isolated NSCLC/CSCs from established cell lines or clinical samples using the expression of CSC markers 22, dye exclusion 23 or spheroid formation [19, 24, 21] as criteria. The isolated CSCs differed markedly in the expression of CSC markers [5, 4], in agreement with our data for H460 and A549 CSCs. Previously isolated CSCs were more resistant to chemotherapy or DNA-damaging treatment 19, also in agreement with our data.

The data discussed above indicate that cisplatin resistance and CSC characteristics could be related properties what prompted us to compare the two independently isolated H460 cell populations. The two populations from both cell types were less sensitive to cisplatin. In addition, CSCs derived from H460R cells presented the same sensitivity to the drug than H460 CSCs indicating that the cisplatin resistance acquired as a consequence of both treatments was not additional. These data indicate that H460C and H460R cells could have acquired similar mechanisms of cisplatin resistance.

H460, H460C, and H460R cell populations generated tumors when subcutaneously injected in immunodeficient mice. However, resistant cells and CSCs generated smaller tumors, with a longer latency period, which could indicate their slower growth. Actually, when mixtures of CSCs and differentiated cells were inoculated, the proportion of CSCs decreased in the tumors generated. However, some proportion of CSCs and differentiated cells showed a high tumorigenic capacity, producing larger tumors, with shorter latency period. The proportion of CSCs increased in these more aggressive tumors. These results indicate that the presence of CSCs and differentiated cells in the same tumor might have synergistic effects and result in the formation of more aggressive tumors, as recently reported in colorectal cancer 25.

CSCs and cisplatin-resistant cells were able to metastasize after intravenous inoculation in immunodeficient mice. Both cell populations produced a large number of metastasis in lungs, as H460 cells did, but also in other organs. CSCs produced a slightly smaller number of metastasis but a combination of CSCs and unselected cells produce a larger proportion, in agreement with the results obtained in the tumorigenic assay. A distinct property of cisplatin-resistant cells was their increased capacity to produce bone metastasis.

Lung cancer bone metastases affect 30–40% of patients with advanced lung cancer and are related to poor prognosis, lower survival time, and skeletal-related events such as pathological fractures, spinal cord compression, hypercalcemia or pain 26. Common bone metastasis sites are pelvis, ribs, vertebral bodies, skull, and the long bones close to the torso 26, being femur a usual location 27 as observed in this work.

Our data correlates with previous observations for highly metastatic lung cancer cell lines 28 where a limited number of metastatic precursors grew within capillaries and extravasation occurred. The subsequent steps were complete colonization of bone marrow and cortical bone lysis 28. Our results (Fig.3) indicate similar behavior for cisplatin-resistant H460 cells. We observed reactive bone formation of murine origin that was fully infiltrated by human tumoral cells.

CSCs and cisplatin-resistant cells also differed in the higher invasive capacity of cisplatin-resistant cells. CSCs, however, showed a higher capacity to induce angiogenesis in immunodeficient mice, which could potentiate tumor growth.

Gene expression analyses also indicated a close relationship between CSCs and cisplatin-resistant H460 cells. Thirteen genes were commonly upregulated, and 13 downregulated, in both cell populations. The differential expression of six of the commonly upregulated genes was confirmed by quantitative RT-PCR. Three of these genes, JUN, EGR1, and AKAP12, are involved in the control of cell proliferation and cancer progression 29. AKAP12 and EGR1expression have been associated with cisplatin resistance 30,31. COX2 (PTGS2) is overexpressed in the first steps of lung carcinogenesis and its overexpression has been considered of bad prognosis 32. COX2 has also been associated with drug resistance in NSCLC 33. MALAT1 codes for a large noncoding RNA and is expressed in tumoral processes, including lung adenocarcinoma 34. The adrenomedullin peptide (ADM) is considered a proto-oncogene that plays multiple roles in cancer 35. ADM induces the expression of early response genes, such as JUN and EGR1 36, angiogenesis and lymphangiogenesis 37. In addition, both COX2 and ADM mediate carcinogenesis produced by cigarette smoke 38,39.

The expression of other genes is specifically regulated in CSCs. Some upregulated genes are involved in cytokine activity, such as IL1A, IL1B, CXCL14, CCL20, AREG, INHBA, and CXCR4 and are repressed upon CSC differentiation. Overexpression of growth factors by CSCs is in agreement with the observation that H460 CSCs proliferate in the absence of added growth factors. Levina et al. 18 also described that CSCs obtained from H460 cells expressed a large number of growth factors and receptors when implanted in SCID mice. The specific growth factors identified in this study do not completely coincide with those found overexpressed in the present article, which could be due to the very different conditions used: Xenografts versus cell culture.

Among these genes IL1B and CXCR4 play an important role in angiogenesis 40, in agreement with the increased angiogenic capacity of CSCs. Previous studies also reported upregulation of CXCR4 in lung CSCs 18 and suggested that this gene plays a role in metastasis and cisplatin resistance 22,41. This molecule has been suggested as a potential target for treatment of metastatic lung cancer 42. LOXL2, upregulated in CSCs, is involved in cell proliferation, metastasis, and angiogenesis 43, and TMEM158 has been related to cisplatin resistance 44.

The genes specifically underexpressed in CSCs are significantly enriched in IGF binding (IGFBP3, 6, 7, NOV, CRIM1) and cell adhesion (ANTXD1, ITGB5, ITGBL1, ITGA7, CDH11, CDH13). CDH13 underexpression was related to tumor invasion and cell migration. Underexpressed endopeptidase inhibitors (SERPIN-B11, -D1) that impair extracellular matrix degradation might result in tumor invasion and metastasis 45.

Resistant cells show significant overexpression of genes involved in the response to chemical stimuli such as five metalothionein-coding genes that confer cisplatin resistance due to their antiapoptotic activity and their capacity to interact with cisplatin 46. In addition, glutation peroxidase 3 (GPX3) has been involved in drug resistance 47.

H460 cisplatin-resistant and CSCs expressed higher levels of DUSP1, DUSP6, and VEGFC genes, previously involved in cisplatin resistance and tumorigenicity 10. Cisplatin-resistant A549 cells also expressed increased levels of these genes while A549 CSCs expressed increased levels of DUSP6 and VEGFC but lower levels of DUSP1.

CSCs from H460 and A549 expressed increased levels of epithelial markers and one mesenchymal marker but decreased levels of a second mesenchymal protein. These changes were reverted upon CSC differentiation. These results are in agreement with recent reports showing that CSC populations present both epithelial and mesenchymal characteristics 48,49.

In summary, H460 CSC and cisplatin-resistant cells have in common their decreased sensitivity to cisplatin, that is not additive in H460R-derived CSCs. Both cell types produce smaller tumors with larger latency periods than untreated cells, indicative of smaller proliferation capacity. A third common characteristic is the similar regulation in the expression of a significant number of genes, Both cell types also differ in some of their properties as are the increased angiogenic capacity of H460C and the larger invasive capacity and production of bone metastasis of H460R cells.

The possible clinical implication of the correlation observed between cisplatin resistance and CSCs has been approached. Forty-four surgical samples of untreated NSCLC patients were analyzed for their sensitivity to cisplatin and for the presence of CSCs. Ten samples were considered sensitive to cisplatin (IC50 < 2 *μ*g/mL) and only one of them was able to grow as spheres in conditioned media. In contrast, 17 of 34 samples resistant to cisplatin generated spheres. Although the absence of CSCs in some samples could be due to the heterogeneity of the tumors and the study of a larger number of patients is required to drive definitive conclusions, statistical analyses of the data obtained indicate that patients who do not host cells able to grow as spheres are more likely to respond to cisplatin treatment (*P* = 0.028).

## Supporting Information

**Table S1.** Tumor formation after subcutaneous injection of H460, H460 CSC (H460C) or cisplatin-resistant (H460R) cells into Nu/Nu mice. **Table S2.** Genes whose expression is regulated in H460 CSC and cisplatin-resistant cells as determined by DNA-microarray hybridization. **Table S3.** Genes whose expression is repressed after the culture of H460 CSC cells under adherent conditions for the periods of time indicated. **Table S4.** General characteristics of the patients from whom biopsies were obtained. **Figure S1.** Characterization of H460 and A549 Cancer Stem Cells. **Figure S2.** Venn diagram comparing the genes regulated in cisplatin-resistant and CSC H460 cells. **Figure S3.** Analysis of differential gene expression by quantitative RT-PCR.
