# Supplementary material for: Cancer stem cells and cisplatin-resistant cells isolated from non-small-lung cancer cell lines constitute related cell populations
Source: Cancer Med. 2014 Jun 25;3(5):1099–111. doi: 10.1002/cam4.291 (PMC4302662; doi:10.1002/cam4.291)
Supplement: Supplementary file 1 [file cam40003-1099-SD1.pdf]

Table S1. Tumour formation after subcutaneous injection of H460, H460 CSC (H460C) or cisplatin resistant (H460R) cells into Nu/Nu mice.

	Cell Number	Latency (days)	Tumour Volume (cm^3)
H460	5000	20 ± 5.1	2.4 ± 0.9
	50000	15 ± 3.22	2.2 ± 1.2
H460C	5000	25 ± 5.82	0.45 ± 0.25
	50000	20 ± 2.21	0.1 ± 0.06
H460R	5000	22 ± 1.41	1.05 ± 0.31
	50000	21 ± 4.18	0.18 ± 0.21

Table S2. Genes whose expression is regulated in H460 CSC and cisplatin-resistant cells as determined by DNA-microarray hybridization.

A. Genes upregulated in cisplatin-resistant cells

**response to chemical stimulus** adjP=1.89e-05	CXCL5; chemokine (C-X-C motif) ligand 5<br>PTGS2; prostaglandin G/H synthase and cyclooxygenase<br>MT1H; metallothionein 1H<br>COLEC12; collectin sub-family member 12<br>GPX3; glutathione peroxidase 3<br>EGR1; early growth response 1<br>PLOD2; procollagen-lysine, 2-oxoglutarate 5-dioxygenase 2<br>IGFBP1; insulin-like growth factor binding protein 1<br>CYR61; cysteine-rich, angiogenic inducer, 61<br>EPS8; epidermal growth factor receptor pathway substrate 8<br>TNFRSF11B; tumor necrosis factor receptor superfamily, member 11b<br>LY96; lymphocyte antigen 96<br>MT1X; metallothionein 1X<br>ADM; adrenomedullin<br>HSPB3; heat shock 27kDa protein 3

B. Genes downregulated in cisplatin-resistant cells:

**L-serine metabolic process** adjP=4.28e-05	CBS; cystathionine-beta-synthase<br>PSAT1; phosphoserine aminotransferase 1<br>PHGDH; phosphoglycerate dehydrogenase
**serine family amino acid biosynthetic process** adjP=6.27e-05	CBS; cystathionine-beta-synthase<br>PSAT1; phosphoserine aminotransferase 1<br>PHGDH; phosphoglycerate dehydrogenase
**cellular amino acid biosynthetic process** adjP=8.91e-05	CBS; cystathionine-beta-synthase<br>ASNS; asparagine synthetase<br>PSAT1; phosphoserine aminotransferase 1<br>PHGDH; phosphoglycerate dehydrogenase

C. Genes upregulated in CSC cells

**sterol biosynthetic process** adjP=8.27e-09	CYP51A1; cytochrome P450, family 51, subfamily A, polypeptide 1<br>LSS; lanosterol synthase (2,3-oxidosqualene-lanosterol cyclase)<br>HMGCS1; 3-hydroxy-3-methylglutaryl-Coenzyme A synthase 1<br>IDI1; isopentenyl-diphosphate delta isomerase 1<br>CS4MOL; sterol-C4-methyl oxidase-like<br>SQLE; squalene epoxidase<br>TM7SF2; transmembrane 7 superfamily

	member 2<br>SCSDL; sterol-C5-desaturase-like<br>EBP; emopamil binding protein (sterol isomerase)
**oxidation reduction** adjP=0.0003	FADS2; fatty acid desaturase 2<br>GPX3; glutathione peroxidase 3<br>SOD2; superoxide dismutase 2<br>TM7SF2; transmembrane 7 superfamily member 2<br>SCD; stearoyl-CoA desaturase<br>LOXL2; lysyl oxidase-like 2<br>SLC1A3; solute carrier family 1 (glial high affinity glutamate transporter), member 3<br>STEAP1; six transmembrane epithelial antigen of the prostate 1<br>CYP51A1; cytochrome P450, family 51, subfamily A, polypeptide 1<br>PTGS2; prostaglandin G/H synthase and cyclooxygenase<br>FA2H; fatty acid 2-hydroxylase<br>CS4MOL; sterol-C4-methyl oxidase-like<br>SQLE; squalene epoxidase<br>IFI30; interferon, gamma-inducible protein 30<br>EGLN3; egl nine homolog 3<br>SCSDL; sterol-C5-desaturase-like
**endopeptidase inhibitor activity** adjP=0.0017	C3; complement component 3<br>SLP1; secretory leukocyte peptidase inhibitor<br>SPINK1; serine peptidase inhibitor, Kazal type 1<br>COL7A1; collagen, type VII, alpha 1<br>SPINK6; serine peptidase inhibitor, Kazal type 6<br>PI3; peptidase inhibitor 3<br>SPINKSL3; serine PI Kazal type 5-like 3
**cytokine activity** adjP=0.0344	CXCL14; chemokine (C-X-C motif) ligand 14<br>IL1A; interleukin 1, alpha<br>CCL20; chemokine (C-C motif) ligand 20<br>AREG; amphiregulin<br>IL1B; interleukin 1, beta<br>INHBA; inhibin, beta A<br>CXCR4; chemokine (C-X-C motif) receptor-4

D. Genes down-regulated in CSC cells

**cell adhesion** adjP=0.0051	CTNNAL1; catenin (cadherin-associated protein), alpha-like 1<br>ERBB2; v-erb-b2 erythroblastic leukemia viral oncogene homolog 2<br>DLC1; deleted in liver cancer 1<br>THBS3; thrombospondin 3

	CDH18; cadherin 18<br>CDH11; cadherin 11<br>ANTXR1; anthrax toxin receptor 1<br>LAMA1; laminin, alpha 1<br>ITGB5; integrin, beta 5<br>APP; amyloid beta (A4) precursor protein<br>SEMASA; semaphorin 5A<br>IGFBP7; insulin-like growth factor binding protein 7<br>PCDH20; protocadherin 20<br>NRXN3; neurexin 3<br>SRPX; sushi-repeat-containing protein, X-linked<br>ITGBL1; integrin, beta-like 1<br>ITGA7; integrin, alpha 7<br>CDH13; cadherin 13, H-cadherin<br>LSAMP; limbic system-associated membrane protein
**insulin-like growth factor binding** adjP=0.0003	NOV; nephroblastoma overexpressed gene<br>IGFBP6; insulin-like growth factor binding protein 6<br>CRIM1; cysteine rich transmembrane BMP regulator 1<br>IGFBP3; insulin-like growth factor binding protein 3<br>IGFBP7; insulin-like growth factor binding protein 7
**calcium ion binding** adjP=0.0007	ANXA3; annexin A3<br>GALNTL1; polypeptide N-acetylgalactosaminyltransferase-like 1<br>PLSCR4; phospholipid scramblase 4<br>KCNIP3; Kv channel interacting protein 3, calsenilin<br>FBN2; fibrillin 2<br>THBS3; thrombospondin 3<br>CDH18; cadherin 18<br>CDH11; cadherin 11<br>FBLN1; fibulin 1<br>CACNA2D3; calcium channel, voltage-dependent, alpha 2/delta subunit 3<br>SLC8A1; solute carrier family 8 (sodium/calcium exchanger), member 1<br>PCDH20; protocadherin 20<br>NRXN3; neurexin 3<br>EFEMP1; EGF-containing fibulin-like extracellular matrix protein 1<br>PXDN; peroxidasin homolog<br>FSTL1; follistatin-like 1<br>PRKCB; protein kinase C, beta<br>ITGA7; integrin, alpha 7<br>FRAS1; Fraser syndrome 1<br>CDH13; cadherin 13<br>MYL5; myosin, light chain 5<br>FKBP10; FK506 binding protein 10

**endopeptidase inhibitor activity**<br>adjP=0.0316	SERPINB11; serpin peptidase inhibitor, clade B (ovalbumin), member 11<br>TIMP2; TIMP metallopeptidase inhibitor 2<br>CRIM1; cysteine rich transmembrane BMP regulator 1<br>APP; amyloid beta (A4) precursor protein<br>SERPNID1; serpin peptidase inhibitor, clade D (heparin cofactor), member 1<br>TFPI; tissue factor pathway inhibitor

Table S3. Genes whose expression is repressed after the culture of H460 CSC cells under adherent conditions for the periods of time indicated.

Time of culture	Repressed genes
3 hours	SOD2; superoxide dismutase 2<br>ANKRD37; ankyrin repeat domain 37
9 hours[a]	CXCR4; chemokine (C-X-C motif) receptor-4<br>IL1A; interleukin 1, alpha<br>IL1B; interleukin 1, beta
24 hours[b]	ADM; adrenomedullin<br>AKAP12; A kinase anchor protein 12<br>AREG; amphiregulin<br>LOXL2; lysyl oxidase-like 2<br>S100A8; calgranulin A<br>IL21R; interleukin 21 receptor<br>EGLN3; egl nine homolog 3

[a] Genes whose expression was found repressed at 3 hours and stayed stable for the following 6 hours (detected after 9 hours).

[a] Genes whose expression was found repressed at 9 hours and stayed stable for the following 15 hours (detected after 24 hours).

Table S4. General characteristics of the patients from whom biopsies were obtained.

Mean age (range)	60.6 (21- 84)
Men Percentage	56.8%
Smoking status	
Ex smoker	18
Current smoker	17
Never smoker	7
Unknown	2
Histology	
Adenocarcinoma	21
Squamous cell carcinoma	14
Large cell carcinoma	3
Other	6
Stage at diagnosis	
I	13
II	17
III	11
IV	2
Unknown	1

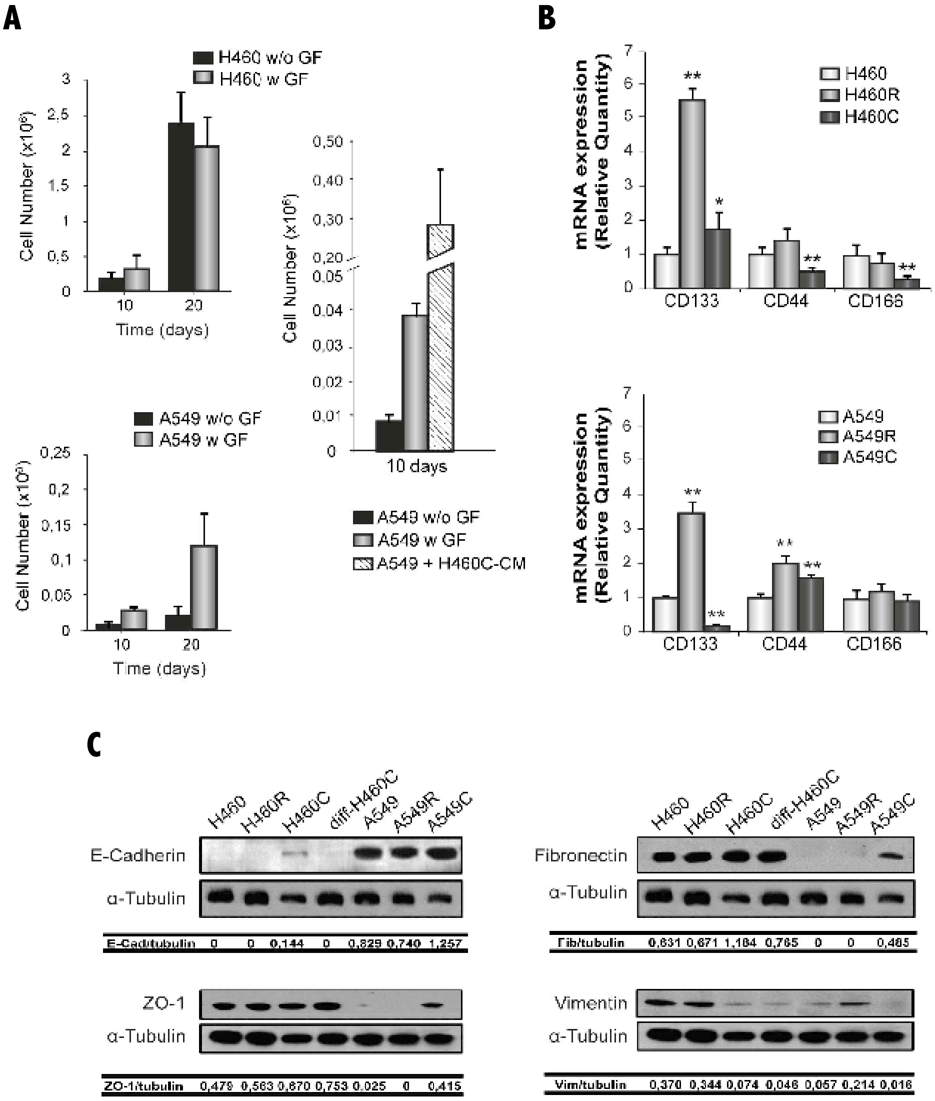

Figure S1. Characterization of H460 and A549 Cancer Stem Cells.
Panel A. Dependence on growth factors. H460 (upper, left panel) and A549 (lower, left panel) cells were incubated for 10 or 20 days under non-adherent conditions in defined media containing the growth factors EGF and bFGF (grey bars) or not (black bars). The number of cells at the end of the incubation was determined. The right panel represents the number of cells obtained after culture of A549 cells in defined media supplement with EGF and bFGF (grey bar), in the presence of H460 conditioned media (H460C-CM, striped bar) or without any addition (black bar) for 10 days. Panel B. The expression of the putative CDC markers CD133, CD44 and CD166 was determined by quantitative RT-PCR. The expression levels in cisplatin-resistant (H460R, A549R) and CSCs (H460C, A549C) were calculated using the Delta Delta CT method, setting the untreated parental cells as endogenous controls with expression levels of one. In panels A and B, averages and standard deviations obtained in three independent experiments are represented $*p<0.05$, $**p>0.01$. Panel C. Analyses of the expression of the epithelial makers E-cadherin, zonula occludentent protein I (ZO) and the mesenchymal markers Fibronectin, Vimentin by Western blot. Extracts were obtained from untreated cells (H460, A549), cisplatin-resistant cells (H460R, A549R), CSCs (H460C, A549C) and CSCs cultured under adherent conditions for 24 hours (Diff-H460C). The expression of α-tubulin was used as loading control. The relative expression of each protein is indicated under the blots.

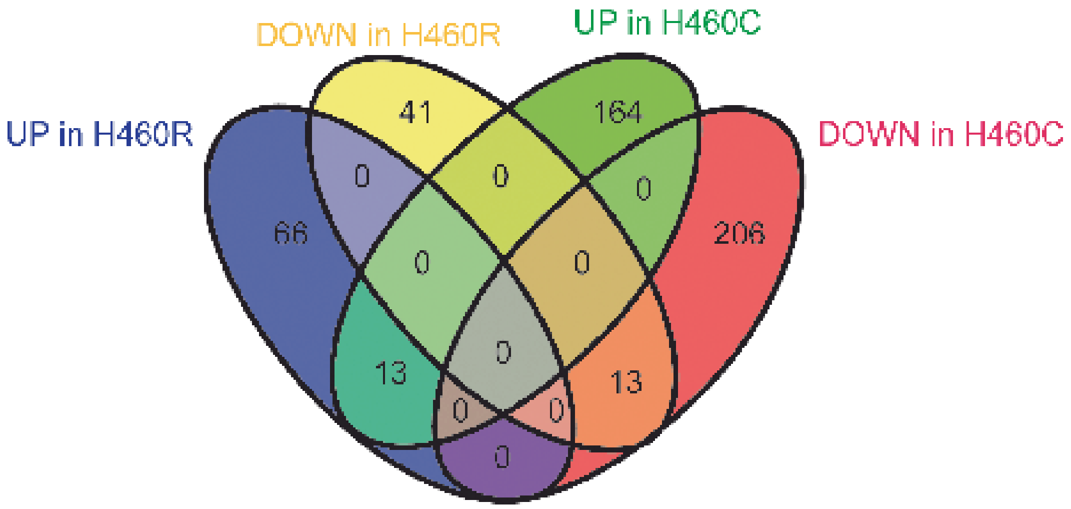

Figure S2. Venn diagram comparing the genes regulated in cisplatin-resistant and CSC H460 cells.
Differential gene expression in Cisplatin-resistant and CSC H460 cells, in relation to untreated H460 cells, was analyzed by DNA microarray hybridization. Up-regulated and Down-regulated genes were compared and the number of genes that are commonly or differentially regulated in both cell populations is shown in the diagram.

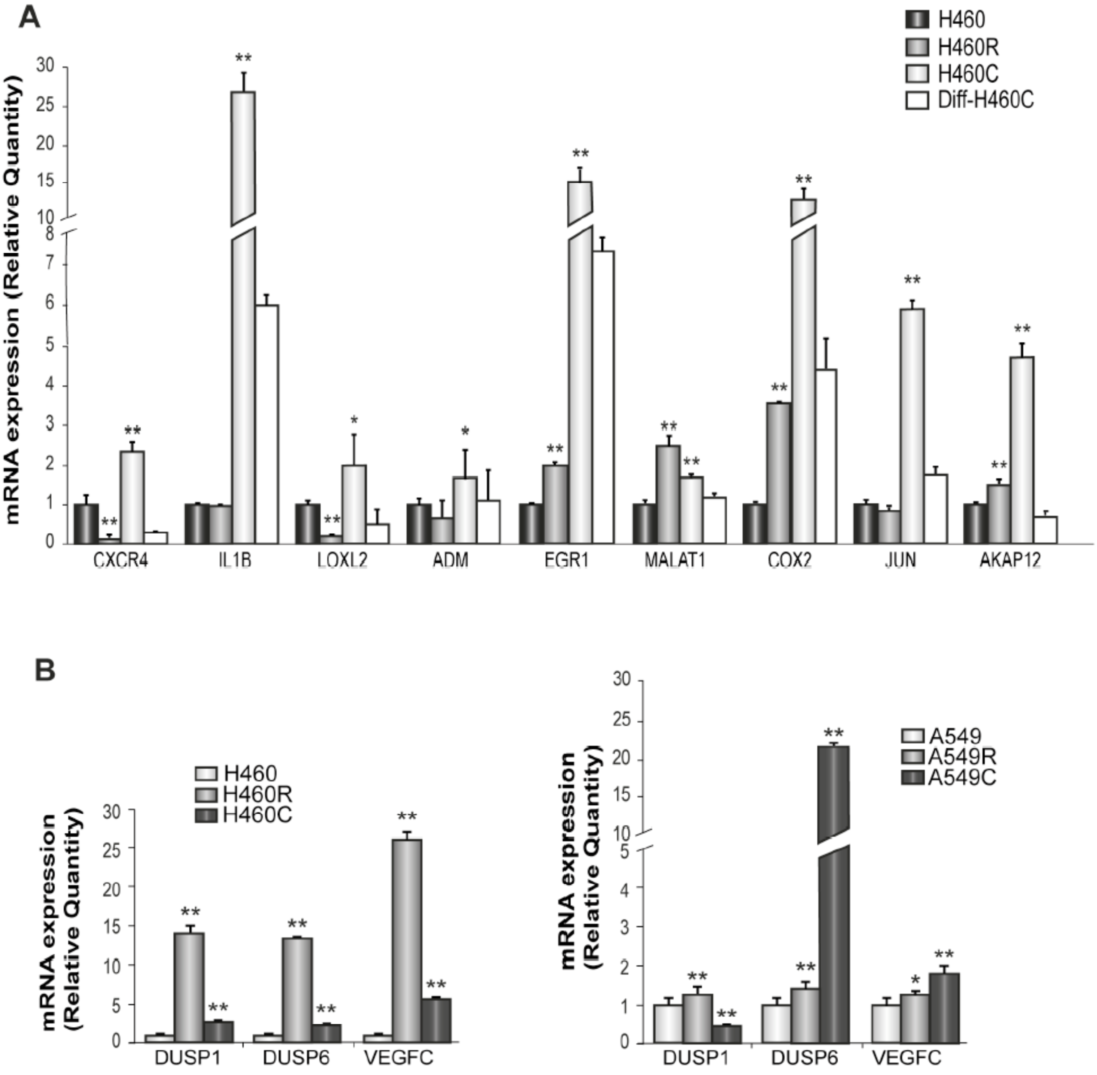

Figure S3. Analysis of differential gene expression by quantitative RT-PCR.
Panel A. The expression of several genes identified by DNA microarray hybridization was further analyzed by quantitative RT-PCR. RNA was isolated from untreated H460 cells (H460), cisplatin-resistant cells (H460R), CSC cells (H460C) and CSCs cultured under adherent conditions for 24 hours (Diff H460C) and converted to cDNA. The expression of the indicated genes was determined by quantitative RT-PCR. Expression levels were calculated using the Delta CT method setting the untreated parental H460 cells as endogenous control with expression levels of one. Panel B. The expression of the DUSP1, DUSP6 and VEGFC genes, previously related with angiogenesis, was determined in untreated (H460, A549), cisplatin-resistant (H460R, A549R) and CSC (H460C, A549C) cells. Expression levels were normalized as described above. Asterisks indicate significant differences in relation to untreated cells in both panels. Average values and standard deviations of two independent experiments are shown *p<0.05, **p>0.01.
